# Inflammatory bowel disease and risk of dementia: An updated meta-analysis

**DOI:** 10.3389/fnagi.2022.962681

**Published:** 2022-10-05

**Authors:** Nanyang Liu, Yi Wang, Lanye He, Jiahui Sun, Xing Wang, Hao Li

**Affiliations:** ^1^Xiyuan Hospital, China Academy of Chinese Medical Sciences, Beijing, China; ^2^Graduate School, Beijing University of Chinese Medicine, Beijing, China; ^3^Wangjing Hospital, China Academy of Chinese Medical Sciences, Beijing, China

**Keywords:** inflammatory bowel disease, dementia, meta-analysis, Alzheimer’s disease, ulcerative colitis, Crohn’s disease

## Abstract

**Background:**

Growing evidence suggests that inflammatory bowel disease (IBD) and dementia share pathological mechanisms and pathogenic risk factors. However, the previously diagnosed IBD and the subsequent risk of developing dementia are largely unknown.

**Aim:**

The purpose of this review is to assess the association between IBD and subsequent dementia diagnosis.

**Methods:**

We searched PubMed, Embase, and Cochrane library from database inception to February 1, 2022. Two reviewers independently extracted data and assessed methodological quality and risk of bias. Observational study that reported the possibility of dementia in IBD and non-IBD populations were included. Eligible studies were pooled effect estimates for relative risk (RR) through fixed-or random-effects models as appropriate.

**Results:**

More than 3,181,549 participants from nine studies met the inclusion criteria. Previous IBD diagnosis did not increased the risk of subsequent all-cause dementia (RR, 1.32; 95% CI, 0.98–1.77) and AD-dementia (RR, 1.62; 95% CI, 0.96–2.76). Subgroup analyses based on study design indicated that cohort studies (RR, 1.30; 95% CI, 1.09–1.55) reported an increased risk of all-cause dementia, but were not applicable to AD-dementia (RR, 1.27; 95% CI, 0.94–1.72). Positive associations between IBD patients and all-cause dementia did not differ by age and gender in cohort studies. Both ulcerative colitis (UC) (RR, 1.39; 95% CI, 1.00–1.94) and Crohn’s disease (RR, 1.46; 95% CI, 1.08–1.98) are associated with increased risk of all-cause dementia.

**Conclusion:**

Evidence regarding dementia risk assessment in IBD patients is conflicting, which may be influenced by study design. More prospective cohort studies are needed to determine their relationship.

**Systematic review registration:**

[https://www.prosper-isd.net], identifier [CRD42021284116].

## Introduction

Inflammatory bowel disease (IBD), mainly including ulcerative colitis (UC) and Crohn’s disease (CD), is characterized by chronic inflammation in the gastrointestinal tract (Graham and Xavier, 2020). The etiology includes multiple risk factors, such as environment, infection, immunity, and genetics (Di Narzo et al., 2021). Gastrointestinal tract inflammation, confirmed by endoscopy and biopsy, usually causes symptoms like abdominal pain, diarrhea, and hematochezia, most of which can be relieved or controlled after the treatment. The interaction between the enteric nervous system and central nervous system, also known as the gut-brain axis, has become a hot topic in recent years. The gut-brain axis involving bidirectional communication between the gut and brain has been confirmed to play important roles in many physiological and pathophysiological processes in the brain (Osadchiy et al., 2019). Neurological complications associated with IBD have been reported. For instance, psychiatric disorders, like depression and anxiety in patients with IBD were significantly more common than those without IBD (Mikocka-Walus et al., 2016). A recent meta-analysis reported a positive association between previously diagnosed IBD and subsequent Parkinson’s disease (PD) risk (Zhu et al., 2022). Additionally, a common structure of genetic variation between IBD and PD was determined in a large-scale genome-wide pleiotropic analysis (Herrick and Tansey, 2021; Lee et al., 2021). Likewise, large-scale Genome-Wide Association Studies demonstrated the genetic correlations and potential causality between multiple sclerosis and each of IBD, UC, and CD (Yang et al., 2021).

Dementia is a progressive neurodegenerative disease that leads to a decline in cognitive ability, Alzheimer’s disease (AD) is the most common type (Liu et al., 2021). Patients are unable to live independently and need long-term care. Accumulating evidence indicates a presence of associations between IBD and dementia (Goyal et al., 2021). Although long-term exposure to IBD has been linked to mild to severe cognitive decline (Hopkins et al., 2021), the role of association with dementia has received little attention. Recently, emerging evidence associates IBD with incident dementia; however, no final conclusions have been drawn on the matter of the relationship between them. The present study, therefore, aims to thoroughly investigate the possible association between IBD and the risk of subsequent dementia.

## Methods

This study has been registered on PROSPERO (CRD42021284116). This study followed the Preferred Reporting Items for Systematic Reviews and Meta-analyses (PRISMA) reporting guideline and the Meta-analysis of Observational Studies in Epidemiology (MOOSE) reporting guideline.

### Search strategy

We searched PubMed, Embase, and Cochrane Library from database inception to October 9, 2021 for articles designed in observational study that reported available data on the risk of dementia in IBD and non-IBD populations. The search strategy included terms related to dementia and IBD, such as Alzheimer’s disease, vascular dementia, dementia with Lewy bodies, Crohn’s disease, and UC. These headings and keywords are combined with the Boolean operators “and” and/or “or.” There are no restrictions on the language of publication. We also performed a gray literature search of the database and checked the references of related studies to obtain other studies that were not identified by electronic search. The detailed search strategy is presented in Supplementary Table 1.

### Study selection and eligibility criteria

Two authors independently reviewed the titles and abstracts, and then screened the full-text articles and cross-checked them. All the discrepancies were resolved through discussion. All eligible studies had to meet the following inclusion criteria: (1) Observational studies with a control group; (2) studies that recorded data on the risk of dementia in patients with IBD and non-IBD. Preclinical studies, case reports, letters to the editor, conference papers, and studies for which the full text was not available were excluded.

### Data extraction

Two authors independently extracted data using a pre-designed extraction form and resolved any discrepancies through discussion. The extracted information included study characteristics (year of publication, region, study design, and follow-up), characteristics of participants (age and gender), selection of cases and controls, diagnosis of IBD and dementia, evaluation of results, i.e., number of participants, odds ratio (OR), relative risk (RR), hazard ratio (HR) reported, and associated raw data for re-calculation or any multivariate analyses adjustment factors (if applicable).

### Quality assessment

Newcastle-Ottawa Scale (NOS) was used to assess the risk of bias for cohort and case-control studies and arbitrarily judged studies with scores greater than 6 had a lower overall risk of bias, (Wells et al., 2018). Cross-sectional studies were assessed *via* the 11-item checklist recommended by the Agency for Healthcare Research and Quality (AHRQ), (Jue et al., 2019).

### Statistical analysis

Meta-analysis was carried out using the STATA metan command. Fixed (Mantel-Haenszel) or random effects (DerSimonian-Laird) models were used to calculate pooled effect estimates. Effect estimates extracted or recalculated from existing data were HRs and ORs. Because of the low absolute risk of dementia, the two measures of association are expected to yield similar RR estimates. Therefore, we pooled all RR estimates appropriately to ensure comprehensiveness of the analysis and maximize statistical power (Siristatidis et al., 2013). The adjusted effect estimates were preferred. Heterogeneity was assessed by using Cochran Q statistic (significance level at *P* < 0.10) and by estimating *I*^2^. Heterogeneity was classified as moderate (*I*^2^ = 25–50%), substantial (*I*^2^ = 50–75%) or considerable (*I*^2^ ≥ 75%). The preplanned subgroup analysis was performed based on gender, study design, and type of disease. Sensitivity analysis of the primary endpoint was conducted by sequential removal of each trial to assess the impact of individual studies on overall pooled estimates. The possibility of publication bias was assessed if sufficient primary studies were included (≥ 10). Variables were compared using χ^2^ tests. All tests were 2-tailed. We considered the *P*-value < 0.05 to be statistically significant.

## Results

**FIGURE 1:**
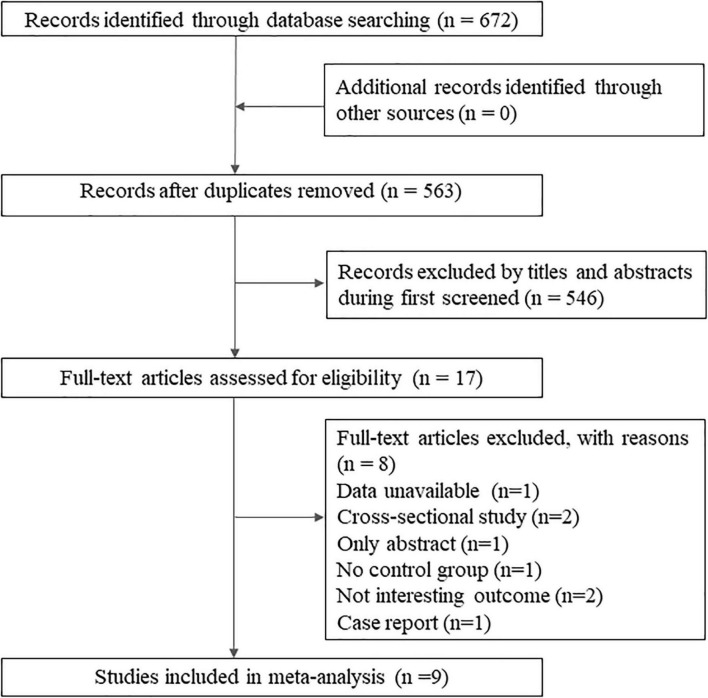
Flow diagram of the literature selection process.

A total of 672 records were identified by database search. After the reviewing of titles and abstracts, 17 articles were retained for full-text reading, and finally, nine studies met the review criteria, including seven cohort studies (Sutton et al., 2019; Bernstein et al., 2021; Kim et al., 2021; Sun et al., 2021; Zhang et al., 2021; Zingel et al., 2021; Sand et al., 2022), one cross-sectional study (Bähler et al., 2017), and one case-control study (Zhou et al.’s, 2020; Figure 1). The detailed exclusion list is provided in Supplementary Table 2.

### Study characteristics

The nine studies totaled more than 3,181,549 participants, including 442, 320 IBD patients and more than 2,739, 229 non-IBD participants. The average age of the participants was over 55 years old. All cohort studies are retrospective, with follow-up ranging from 8 to 31 years. The diagnosis of dementia and IBD was based on the International Classification of Diseases (version 9 or 10) classification code. Eight studies adjusted for confounders, generally covering age, gender, and IBD type. The population characteristics of the included studies are summarized in Table 1.

**TABLE 1:** The population characteristics of the included study.

Source year	Country	Study design	Sample size		Age (M ± SD)		Participant		Follow-up time (year)	Diagnosis of IBD/dementia	Adjustment factor
								
			Case	Control	Case	Control	Case	Control			
Bähler et al. (2017)	Switzerland	Cross-sectional study	4,791	111,4638	56 (28)^a^	44 (37)^a^	IBD	Patients without IBD	NA	NR	Age, sex, language area, type of insurance coverage, and type of residence using entropy balancing
Bernstein et al. (2021)	Canada	Retrospective cohort study	9,247	85,691	NR	NR	IBD (UC and CD)	Patients without IBD were matched to IBD patients (1:10) by age, sex, and geographic residence by postal forward sortation area	1984–2018	ICD-9 ICD-10	Age, sex
Kim et al. (2021)	South Korea	Retrospective cohort study	24,830	99,320	55.4 ± 11.0	55.4 ± 11.0	IBD (UC and CD)	Patients without IBD	2009–2017	ICD-10	Age, sex, residential area, and co-morbidities
Sand et al. (2022)	Denmark	Retrospective cohort study	88,985	884,108	NA	NA	IBD (UC and CD)	Patients without IBD were matched to IBD patients (1:10) by age, sex, and region of residence.	1977–2018	NA	Charlson Comorbidity Index Score, chronic obstructive pulmonary disease (as a proxy for smoking), atrial fibrillation or flutter, diabetes (type 1 and 2), depression, and head trauma
Sutton et al. (2019)	United States	Retrospective cohort study	24,057	42,205	66.7 ± 8.03	66.04 ± 7.89	IBD (UC and CD)	Patients without IBD	2000–2018	ICD-9 ICD-10	NR
Sun et al. (2021)	China	Prospective cohort study	5,778	491,997	57.0 ± 8.1	57.5 ± 8.0	IBD (UC and CD)	Patients without IBD	2010–2021	ICD-9 ICD-10	Age, age-square, sex, race, education, Townsend deprivation index, body mass index, alcohol drinker status, smoking status, physical activity, family history of dementia
Zingel et al. (2021)	Germany	Retrospective cohort study	3,850	3,850	70.9 ± 7.3	71.0 ± 7.2	IBD (UC and CD)	Patients without IBD were matched to IBD patients (1:1) based on age group, gender, index year, health insurance type and comorbidities, hypertension, stroke including transient ischemic attack and depression	1995–2014	ICD-10	IBD type, gender, and age
Zhang et al. (2021)	China	Retrospective cohort study	1,742	17,420	60.64 ± 10.75	60.63 ± 10.76	IBD (UC and CD)	Patients without IBD were matched to IBD patients (1:10) based on age (±1 year), sex, enrolment time, dementia-related medical comorbidities, income level, and urbanization level of residence	1995–2010	ICD-9	Age, charlson comorbidity index score and all-cause clinical visits
Zhou et al.’s (2020)	United States	Case-control study	279,040 NR		>18 years old		Inflammatory disease	Non-inflammatory disease	NA	SNOMED-CT disease names	Age, gender, race, and body mass index, smoking status, or alcohol use

CD, Crohn’s disease; IBD, inflammatory bowel disease; ICD, international classification of diseases; NA, not applicable; NR, not reported; NR, not report; M ± SD; mean standard deviation; SNOMED-CT, systematized nomenclature of medicine-clinical terms; UC, ulcerative colitis.

^a^Median (IQR).

### Risk of bias assessment

Quality assessment scores using the NOS tool range from 7 to 9 stars (Supplementary Table 3). All studies were considered at low risk of bias according to eligibility criteria. In terms of case selection, Zhou et al.’s (2020) study did not define the source of cases in the control group. In terms of comparability, Sutton et al. (2019) received one star for not adjusting for other confounders. For exposure/outcome, the study by Bernstein et al. (2021) did not provide follow-up data, and the Zhou et al.’s (2020) did not provide information on response rates. Quality assessment of the cross-sectional study using the AHRQ yielded 5 “yes,” which was considered moderate risk (Supplementary Table 4).

### Meta-analysis of the association between inflammatory bowel disease and dementia

#### All-cause dementia

**FIGURE 2:**
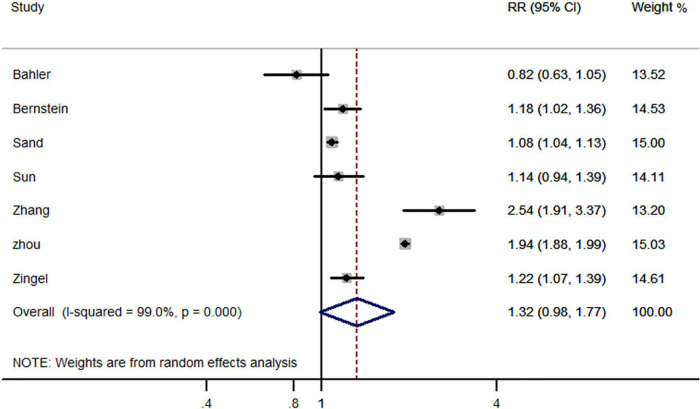
Forest plot presenting effect estimates for the risk of developing all-cause dementia in patients with inflammatory bowel disease.

Seven studies reported all-cause dementia risk in IBD and non-IBD populations, including five cohort studies, one case-control study and one cross-sectional study. Pooled effect estimates suggest that previously diagnosed IBD did not increase the risk of subsequent all-cause dementia (RR, 1.32; 95% CI, 0.98–1.77) (Figure 2).

**FIGURE 3:**
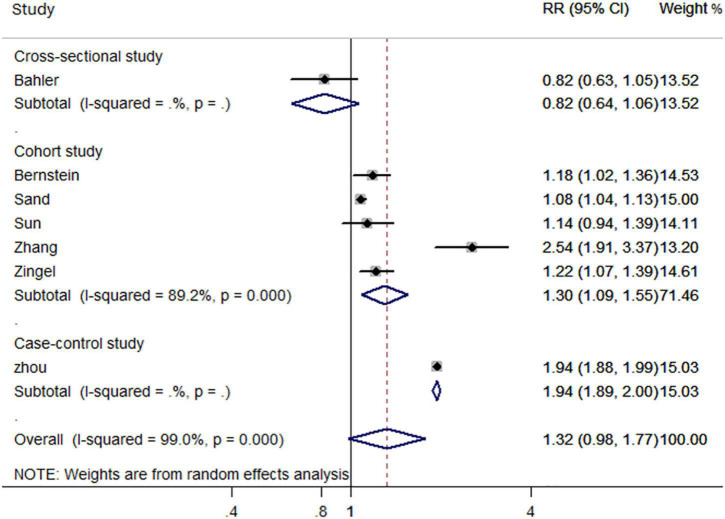
Forest plot presenting study design-based analysis of effect estimates for the risk of developing all-cause dementia in patients with inflammatory bowel disease.

In the subgroup analysis based on study design, cohort studies (RR, 1.30; 95% CI, 1.09–1.55) and case-control studies (RR, 1.94; 95% CI, 1.89–2.00) reported an increased risk of all-cause dementia in patients with IBD. However, the only cross sectional study did not support such an association (RR, 0.82; 95% CI, 0.64–1.06) (Figure 3). Additionally, gender-based subgroup analysis indicated that IBD was associated with an increased risk of all-cause dementia in males (RR, 1.36; 95% CI, 1.00–1.85) and females (RR, 1.38; 95% CI-1.10, 1.72) (Table 2). Further stratified analysis did not support age-related all-cause dementia risk in IBD patients (Table 2).

**TABLE 2:** Results of meta-analysis of the association between IBD and dementia.

Subgroup	Studies (*n*)	RR	95% CI	*P*-value	Heterogeneity (*I*^2^, *P*-value)
** All-cause dementia**					
**IBD**					
** Age**					
< 65 Years old	2	1.33	1.03–1.72	0.061	33.0%, 0.222
≥ 65 Years old	2	1.12	1.00–1.25	0.061	0.0%, 0.582
UC	6	1.39	1.00–1.94	0.048	98.9%, < 0.001
Cohort study	5	1.25	1.03–1.51	0.022	86.2%, < 0.001
Case-control study	1	1.94	1.89–2.00	<0.001	NA
CD	6	1.46	1.08–1.98	0.013	96.6%, < 0.001
Cohort study	5	1.30	1.10–1.54	0.002	62.2%, 0.032
Case-control study	1	2.00	1.94–2.06	<0.001	NA
**AD-dementia**					
UC	4	1.57	1.10–2.25	0.014	98.2%, < 0.001
Cohort study	3	1.25	0.99–1.60	0.064	87.9%, < 0.001
Case-control study	1	1.82	1.74–1.91	<0.001	NA
CD	4	1.78	0.98–3.22	0.059	98.0%, < 0.001
Cohort study	3	1.38	0.86–2.20	0.182	89.0%, < 0.001
Case-control study	1	2.33	2.23–2.44	<0.001	NA

AD, Alzheimer’s disease; CD, Crohn’s disease; IBD, inflammatory bowel disease; NA, not applicable; RR, relative risk; UC, ulcerative colitis.

We also pooled the risk estimates of IBD types and found that both UC (RR, 1.39; 95% CI, 1.00–1.94) and CD (RR, 1.46; 95% CI, 1.08–1.98) were positively associated with the risk of all-cause dementia. These associations remained statistically significant when stratified by study type (Table 2).

#### Alzheimer’s disease-dementia

**FIGURE 4:**
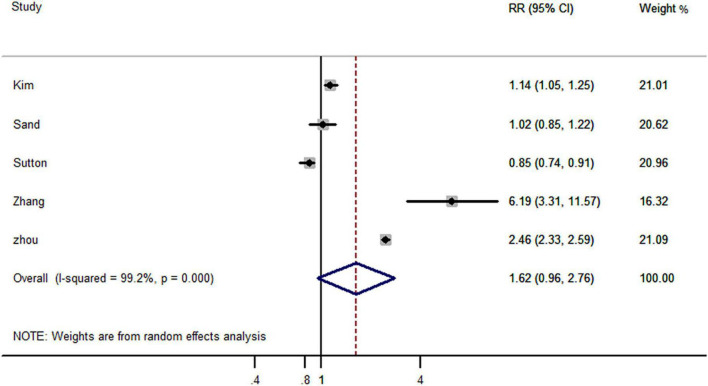
Forest plot presenting effect estimates for the risk of developing AD dementia in patients with inflammatory bowel disease.

Five studies reported the risk of AD-dementia in IBD and non-IBD populations, including four cohort studies and one case-control study. The results showed that previously diagnosed IBD did not increased the risk of subsequent AD-dementia (RR, 1.62; 95% CI, 0.96–2.76) (Figure 4).

**FIGURE 5:**
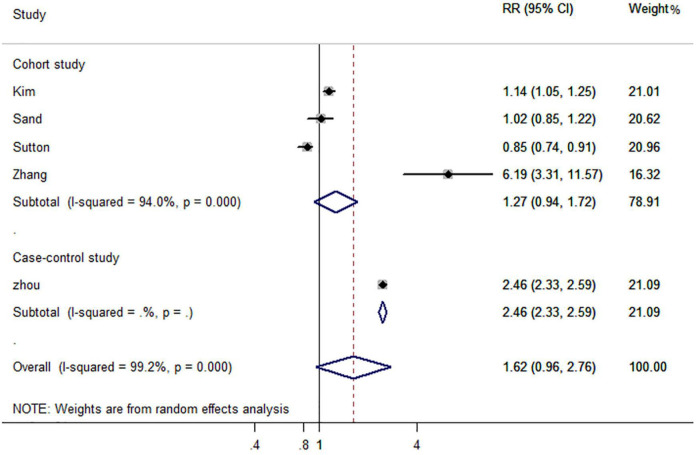
Forest plot presenting study design-based analysis of effect estimates for the risk of developing all-cause dementia in patients with inflammatory bowel disease.

In subgroup analyses based on study design, the case-control study (RR, 2.46; 95% CI, 2.33–2.59) reported an increased risk of AD dementia, but this was not applicable to cohort studies (RR, 1.27; 95% CI, 0.94–1.72) (Figure 5). Interestingly, gender-based stratification showed that IBD did not increase significantly the risk of dementia neither in males (RR, 2.14; 95% CI, 0.51–9.01) nor in females (RR, 2.85; 95% CI, 0.45–18.10) gender (Table 2).

For stratified analysis by IBD type, pooled effect estimates supported a positive association between UC and AD-dementia (RR, 1.57; 95% CI, 1.10–2.25), but not CD (RR, 1.78; 95% CI, 0.98–3.22). However, when stratified by study type, pooled results from cohort studies showed that neither UC (RR, 1.25; 95% CI-0.99, 1.60) nor CD (RR, 1.38; 95% CI, 0.86–2.20) increased the risk of AD-dementia (Table 2).

#### Sensitivity analysis and publication bias

Significant heterogeneity was found for all comparison groups. Sensitivity analysis was performed by removing each study in turn and reanalyzing the dataset, which did not result in a substantial change in risk estimates. Since none of the comparison groups had more than 10 primary studies, publication bias was not assessed.

## Discussion

We performed a systematic review and meta-analysis to comprehensively investigate the risk of dementia in IBD patients. The findings, including more than 3,181,549 participants, suggest that the previous IBD diagnosis did not increased the risk of subsequent all-cause dementia and AD-dementia. However, in the subgroup analysis based on the study design we found an increased risk of all-cause dementia but not AD dementia in IBD patients in the cohort study. Interestingly, UC increased the risk of subsequent all-cause dementia and AD dementia, whereas CD only increased the risk of all-cause dementia.

Our findings are somewhat inconsistent compared with previously published meta-analyses. The study by Zuin et al. (2022) included three cohort studies showing an increased risk of subsequent dementia in IBD patients (HR, 1.52; 95% CI 1.04–2.02). Subjects with CD showed that dementia with an HR of 1.48 (95% CI 1.07–2.03), whereas subjects with UC did not reach the statistical significance (HR, 1.47; 95% CI 0.95–2.82). In addition, male with IBD have an increased risk of dementia compared with female. A meta-analysis by Zhang et al. (2022) including seven primary studies, found that IBD patients had a significantly higher overall risk of all-cause dementia and AD dementia than the general population. However, this study was not stratified by study type, possibly overstating the validity of the results. Another meta-analysis pooling 6 primary studies showed a significantly increased risk of dementia after an IBD diagnosis, regardless of age, gender, dementia subtype, or IBD subtype (Liu et al., 2022). However, the authors did not account for all-cause dementia and AD dementia when pooling effect estimates. Our work included nine primary studies demonstrating that pooled RR effect estimates did not reach statistical significance for either all-cause dementia or AD dementia. However, when grouped by study design and IBD type, some meaningful results were found. Furthermore, the risk of developing dementia in people with IBD does not vary by age and gender.

Several additional studies were not included in the meta-analysis. A cohort study by Caini et al. (2016) found that the risk of UC patients dying from AD was higher than the general population. Papathanasiou et al. (2014) reported two cases of rapidly progressive dementia as presenting feature in patients with CD. One of them was a 56-year-old woman with a Mini-Mental Status Examination (MMSE) score of 17/30, exhibiting episodic abdominal pain, chronic constipation, and moderate to significant apathy. Her cognitive function improved rapidly after being treated with 60 mg/d prednisolone. During the 6-month follow-up, there was no recurrence of symptoms, with an MMSE score of 30/30. Another 50-year-old man was hospitalized with rapidly progressive dementia (MMSE score of 10/30) caused by CD (highly suspected). The cognitive function improved quickly after taking corticosteroid hormones. This was likely a rare case of reversible cognitive impairement due to other causes rather than to a neurodegenerative condition. Li (2017) investigated the prevalence of dementia in 788,103 patients with autoimmune diseases. After adjusting for age, gender, and comorbidities, UC was found to be high-risk for all-cause dementia and AD. This study was not considered for analysis due to the lack of non-IBD groups.

Although there is no clear evidence to support IBD as a risk factor for dementia, we cannot rule out this possibility. The following explanations can illustrate their potential relationship. One explanation is linked to systemic inflammation. AD is the most common type of dementia, which is characterized by amyloid plaques, neurofibrillary tangles, and neuroinflammation (Busche et al., 2019). Studies have shown that neuroinflammation triggered by the brain’s immune system can drive tau hyperphosphorylation and amyloid β accumulation in neurons (Qian et al., 2021). IBD is a chronic inflammatory disease that affects the intestines and parenteral areas. Although the deep remission has been achieved, histological activity and extra-intestinal symptoms may continue to exist, leading to the systemic inflammatory burden, thereby possibly triggering extraintestinal complications, like neurodegenerative diseases. A recent study investigated the influence of increased gut inflammation on brain pathology in the AD model (Sohrabi et al., 2021). Amyloid precursor protein transgenic mice induced by dextran sulfate sodium developed an IBD-like condition. Brain histological and biochemical evaluations demonstrated increased insoluble amyloid β 1–40/42 levels along with the decreased microglial CD68 immunoreactivity in transgenic mice. The key point of the second explanation is the damaged intestinal barrier (also known as intestinal leakage). The IBD-like condition allows intestinal epithelial mucosal damage including physical and biochemical barriers (Sohrabi et al., 2021). Toxic substances (even inflammatory factors) produced by intestinal microorganisms that enter the blood circulatory system affected by intestinal leakage, and then enter the brain through the blood-brain barrier, which, in turn, could contribute to neuroinflammation and neurodegeneration in the central nervous system (Lavelle and Sokol, 2020). Carloni et al. (2021) uncovered that the inflammatory process of IBD makes the gut vascular barrier more permeable, causing inflammation to spread beyond the intestine. Although the vascular barrier in the choroid plexus is closed, helping protect the brain from inflammation, it may also impair communication between organs and impair some brain functions. Another explanation is due to gut dysbiosis. Accumulating evidence indicates a presence of pathogenetic associations between gut dysbiosis and IBD. Alterations in the composition and function of the microbiota have been described in many studies on IBD. Specific classes of metabolites, notably bile acids, short-chain fatty acids, and tryptophan metabolites, are thought to be involved in the IBD pathology (Lavelle and Sokol, 2020). This dysbiosis has also been emphasized to be associated with AD pathology (Kim et al., 2020; Shukla et al., 2021). Gut dysbiosis causes an increase in the number of pro-inflammatory cytokines and a decrease in anti-inflammatory bacteria, ultimately affecting central function through the gut-brain axis (Kim et al., 2020). Overall, gut dysbiosis and the impaired intestinal epithelial barrier associated with IBD may promote the direct entry of neurotoxic metabolites and even bacteria derived from intestinal microorganisms into the brain.

Several limitations need to be addressed. First, retrospective observational studies were included, and most participants were recruited from Medicare databases or hospitalization registries. Therefore, differences in disease diagnosis and data integrity may affect the results. Second, most studies lack complete demographic information, failing to consider other potential confounding factors that affect dementia, such as diet, exercise, mental health, and drug use. Therefore, further research is needed to include more comprehensive demographic data on other confounding factors. Third, limited primary study, especially in subgroup analysis, led to insufficient statistical strength. Fourth, results from epidemiological studies suggest potential associations but are still insufficient to confirm causality. Further studies are needed to verify the causality between them, such as large-scale prospective studies and randomized controlled trials. Fifth, significant heterogeneity may confuse current results. However, we used a random-effects model to pool effect estimates, which can truly reflect the degree of deviation of estimates between studies to obtain more conservative results. Sixth, peripherally acting anticholinergics used for IBS can worsen dementia as well, possibly due to increased use prior to IBD diagnosis, while detailed documentation of this information may be lacking in electronic medical records.

## Conclusion

This meta-analysis underlines that study design may have influenced the assessment of dementia risk in IBD patients and more prospective cohort studies are needed to determine their relationship. However, although controversial, these findings highlight the possibility that IBD is associated with subsequent dementia development and the importance of increased clinical vigilance and early active interventions for this population.

## Data availability statement

The original contributions presented in this study are included in the article/Supplementary material, further inquiries can be directed to the corresponding authors.

## Author contributions

NL, XW, LH, and HL designed the study. JS performed literature search. NL and YW analyzed the data and drafted the first version of the manuscript. XW revised the manuscript. All authors interpreted the data, contributed substantially to the manuscript, and approved the final manuscript for submission, were responsible for the integrity, accuracy, and presentation of the data.

## Abbreviations

AHRQ: Agency for Healthcare Research and Quality
AD: Alzheimer’s disease
CD: Crohn’s disease
CI: confidence interval
IBD: inflammatory bowel disease
MMSE: Mini-Mental Status Examination
NOS: Newcastle-Ottawa Scale
ORs: odds ratios
UC: ulcerative colitis
PD: Parkinson’s disease.
