# Supplementary material for: Inflammatory bowel disease and risk of dementia: An updated meta-analysis
Source: Front Aging Neurosci. 2022 Oct 5;14:962681. doi: 10.3389/fnagi.2022.962681 (PMC9581261; doi:10.3389/fnagi.2022.962681)
Supplement: Supplementary file 1 [file Data_Sheet_1.docx]

**Supplementary materials**

Table 1. Detailed search query.

Table 2. Excluded articles at full-text assessment.

Table 3. Assessment according to Newcastle-Ottawa Rating Scale.

Table 4. Assessment according to Agency for Healthcare Research and Quality.

| **Table 1. Detailed search query** |
| --- |
| **Database:** PubMed  **Date limits:** From database inception to February 1, 2022 |
| Dementia [MeSH] OR Dementia [Abti] OR Dementias [Abti] OR Amentia [Abti] OR Amentias [Abti] OR Alzheimer Dementia [Abti] OR Alzheimer Dementias [Abti] OR Dementia, Alzheimer [Abti] OR Alzheimer's Disease [Abti] OR Alzheimer's Disease [MeSH] OR Dementia, Senile [Abti] OR Senile Dementia [Abti] OR Dementia, Alzheimer Type [Abti] OR Alzheimer Type Dementia [Abti] OR Dementia, Alzheimer-Type [Abti] OR Alzheimer Type Senile Dementia [Abti] AND Inflammatory Bowel Disease [MeSH] OR Inflammatory Bowel Disease [Abti] OR Bowel Diseases, Inflammatory [Abti] OR Crohn's Enteritis [Abti] OR Regional Enteritis [Abti] OR Crohn's Disease [Abti] OR Crohns Disease [Abti] OR Ulcerative colitis [Abti] |
| Database: Embase  **Date limits:** From database inception to February 1, 2022 |
| “dementia” exp/OR “dementia” ab ti: OR “dementias” ab ti: OR “alzheimer dementia”/exp OR “alzheimer dementia” ab ti: OR “alzheimer dementias” ab ti: OR “dementia, alzheimer” ab ti: OR “alzheimer's disease” ab ti: OR “dementia, Senile” ab ti: OR “senile dementia” ab ti: OR “dementia, alzheimer type” ab ti: OR “alzheimer type dementia” ab ti: OR “alzheimer-type dementia” ab ti: OR “alzheimer type dementia” ab ti: OR “dementia, alzheimer-type” ab ti: OR “alzheimer type senile dementia” ab ti: AND “inflammatory bowel disease”/exp OR “inflammatory bowel disease” ab ti: OR “bowel diseases, inflammatory” ab ti: OR “crohn's enteritis” ab ti: OR “regional enteritis” ab ti: OR “crohn's disease” /exp OR “crohn's disease” ab ti: OR “crohns disease” / exp “crohns disease” OR ab ti: OR “ulcerative colitis” ab ti |
| **Database:** Cochrane library  **Date limits:** From database inception to February 1, 2022 |
| Dementia [Abtikw] OR Dementias [Abtikw] OR Amentia [Abtikw]OR Amentias [Abtikw] OR Alzheimer Dementia [Abtikw] OR Alzheimer Dementias [Abtikw] OR Dementia, Alzheimer [Abtikw] OR Alzheimer's Disease [Abtikw] OR Dementia, Senile [Abtikw] OR Senile Dementia [Abtikw] OR Dementia, Alzheimer Type [Abtikw] OR Alzheimer Type Dementia [Abtikw] OR Alzheimer-Type Dementia (ATD) [Abtikw] OR Alzheimer Type Dementia (ATD) [Abtikw] OR Dementia, Alzheimer-Type (ATD) [Abtikw] OR Alzheimer Type Senile Dementia [Abtikw] AND Inflammatory Bowel Disease [Abtikw] OR Bowel Diseases, Inflammatory [Abtikw] OR Crohn's Enteritis [Abtikw] OR Regional Enteritis [Abtikw] OR Crohn's Disease [Abtikw] OR Crohns Disease [Abtikw] OR Ulcerative colitis [Abtikw] |

| **Table 2. Excluded articles at full-text assessment** | |
| --- | --- |
| **First author** | **Reason of exclusion** |
| Heikkila et al^1^ | Data unavailable |
| Crawford et al^2^ | Only abstract |
| Li et al^3^ | No control group |
| Caini et al^4^ | Not interesting outcome (investigating the mortality of IBD patients) |
| Papathanasiou et al^5^ | Case report |
| Cooke et al^6^ | Cross-sectional study |
| Zhu et al^7^ | Not interesting outcome (Parkinson's disease) |
| Santiago et al^8^ | Not interesting outcome (review) |

| **Table 3 Assessment according to Newcastle-Ottawa Rating Scale** | | | | | |
| --- | --- | --- | --- | --- | --- |
| **Source** | **Study design** | **Selection** | **Comparability** | **Exposure/Outcome** | **Total** |
| Bernstein  et al | Retrospective cohort study | **** | ** | ** | 8 |
| Kim  et al | Retrospective cohort study | **** | ** | *** | 9 |
| Sand  et al | Retrospective cohort study | *** | ** | ** | 7 |
| Sutton  et al | Retrospective cohort study | **** | * | *** | 8 |
| Sun  et al | Prospective cohort study | **** | ** | *** | 9 |
| Zingel  et al | Retrospective cohort study | **** | ** | *** | 9 |
| Zhang  et al | Retrospective cohort study | **** | ** | *** | 9 |
| Zhou  et al | Case-control  study | *** | ** | ** | 7 |

| **Table 4 Assessment according to Agency for Healthcare Research and Quality** | |
| --- | --- |
| Source | Bähler  et al |
| Study design | Cross-sectional study |
| Agency for Healthcare Research and Quality list |  |
| Define the source of information (survey, record review) | Yes |
| List inclusion and exclusion criteria for exposed and unexposed subjects (cases and controls) or refer to previous publications | Yes |
| Indicate time period used for identifying patients | No |
| Indicate whether or not subjects were consecutive if not population-based | Yes |
| Indicate if evaluators of subjective components of study were masked to other aspects of the status of the participants | not clear |
| Describe any assessments undertaken for quality assurance purposes (e.g., test/retest of primary outcome measurements) | Yes |
| Explain any patient exclusions from analysis | Yes |
| Describe how confounding was assessed and/or controlled. | Yes |
| If applicable, explain how missing data were handled in the analysis | NA |
| Summarize patient response rates and completeness of data collection | No |
| Clarify what follow-up, if any, was expected and the percentage of patients for which incomplete data or follow-up was obtained | No |

**References:**

**1**. Heikkila T, Tolppanen AM, Koponen M, Hartikainen S, Tiihonen M. Inflammatory bowel disease medication use among community-dwelling persons with and without Alzheimer's disease. *PHARMACOEPIDEM DR S*. 2020;29(SUPPL 3):290. doi:10.1002/pds.5114

**2**. Crawford B, Tomari A. Prevalence of gastrointestinal events in patients with alzheimer's disease in Japan. *VALUE HEALTH*. 2017;20(5):A187.

**3**. Li X. Dementia and Alzheimer's disease risks in autoimmune disorders patients. *NEURODEGENER DIS*. 2017;17(1706.

**4**. Caini S, Bagnoli S, Palli D, et al. Total and cancer mortality in a cohort of ulcerative colitis and Crohn's disease patients: The Florence inflammatory bowel disease study, 1978–2010. *DIGEST LIVER DIS*. 2016;48(10):1162-1167. doi:10.1016/j.dld.2016.07.008

**5**. Papathanasiou A, Nikakis P, Bonakis A, et al. Rapidly progressive dementia as presenting feature in inflammatory bowel disease. *ALZ DIS ASSOC DIS*. 2014;28(3):294-295. doi:10.1097/WAD.0b013e31826a96b2

**6**. Cooke WT, Mallas E, Prior P, Allan RN. Crohn's disease: Course, treatment and long term prognosis. *Quarterly journal of medicine*. 1980;49(195):363.

**7**. Zhu Y, Yuan M, Liu Y, et al. Association between inflammatory bowel diseases and Parkinson's disease: Systematic review and meta-analysis. *NEURAL REGEN RES*. 2022;17(2):344-353. doi:10.4103/1673-5374.317981

**8**. Santiago JA, Potashkin JA. The impact of disease comorbidities in alzheimer's disease. *FRONT AGING NEUROSCI*. 2021;13(631770. doi:10.3389/fnagi.2021.631770
